# Supplementary material for: Pyruvate Dehydrogenase Kinase 1 inhibition mediated oxidative phosphorylation enhancement in cartilage promotes osteoarthritis progression
Source: BMC Musculoskelet Disord. 2023 Jul 20;24:597. doi: 10.1186/s12891-023-06585-6 (PMC10357736; doi:10.1186/s12891-023-06585-6)
Supplement: Supplementary file 1 — Additional file 1. [file 12891_2023_6585_MOESM1_ESM.zip › MF.Gsea.1653627316242/GOMF_TRANSCRIPTION_COACTIVATOR_BINDING.html]

Details for gene set GOMF\_TRANSCRIPTION\_COACTIVATOR\_BINDING[GSEA]

|  || Dataset | 55457\_collapsed\_to\_symbols.cls.cls #OA\_versus\_normal.cls.cls #OA\_versus\_normal\_repos |
| Phenotype | cls.cls#OA\_versus\_normal\_repos |
| Upregulated in class | normal |
| GeneSet | GOMF\_TRANSCRIPTION\_COACTIVATOR\_BINDING |
| Enrichment Score (ES) | -0.51707596 |
| Normalized Enrichment Score (NES) | -1.8647989 |
| Nominal p-value | 0.0 |
| FDR q-value | 0.047576442 |
| FWER p-Value | 0.301 |
Table: GSEA Results Summary

  

Fig 1: Enrichment plot: GOMF\_TRANSCRIPTION\_COACTIVATOR\_BINDING      
 Profile of the Running ES Score & Positions of GeneSet Members on the Rank Ordered List

  

| SYMBOL | TITLE | RANK IN GENE LIST | RANK METRIC SCORE | RUNNING ES | CORE ENRICHMENT || 1 | ESR1 | estrogen receptor 1 [Source:HGNC Symbol;Acc:HGNC:3467] | 984 | 0.522 | -0.0380 | No |
| 2 | MED6 | mediator complex subunit 6 [Source:HGNC Symbol;Acc:HGNC:19970] | 1744 | 0.393 | -0.0681 | No |
| 3 | SIX1 | SIX homeobox 1 [Source:HGNC Symbol;Acc:HGNC:10887] | 2011 | 0.360 | -0.0622 | No |
| 4 | NFATC1 | nuclear factor of activated T cells 1 [Source:HGNC Symbol;Acc:HGNC:7775] | 3027 | 0.247 | -0.1229 | No |
| 5 | RELA | "RELA proto-oncogene, NF-kB subunit [Source:HGNC Symbol;Acc:HGNC:9955]" | 3942 | 0.161 | -0.1821 | No |
| 6 | SMAD3 | SMAD family member 3 [Source:HGNC Symbol;Acc:HGNC:6769] | 4061 | 0.150 | -0.1802 | No |
| 7 | STAT1 | signal transducer and activator of transcription 1 [Source:HGNC Symbol;Acc:HGNC:11362] | 4495 | 0.113 | -0.2055 | No |
| 8 | GATA6 | GATA binding protein 6 [Source:HGNC Symbol;Acc:HGNC:4174] | 5537 | 0.030 | -0.2843 | No |
| 9 | TERT | telomerase reverse transcriptase [Source:HGNC Symbol;Acc:HGNC:11730] | 5773 | 0.010 | -0.3018 | No |
| 10 | CCNT2 | cyclin T2 [Source:HGNC Symbol;Acc:HGNC:1600] | 8192 | -0.170 | -0.4772 | No |
| 11 | HAND2 | heart and neural crest derivatives expressed 2 [Source:HGNC Symbol;Acc:HGNC:4808] | 8211 | -0.172 | -0.4659 | No |
| 12 | AR | androgen receptor [Source:HGNC Symbol;Acc:HGNC:644] | 8217 | -0.173 | -0.4536 | No |
| 13 | SMAD4 | SMAD family member 4 [Source:HGNC Symbol;Acc:HGNC:6770] | 9035 | -0.235 | -0.4998 | Yes |
| 14 | PGR | progesterone receptor [Source:HGNC Symbol;Acc:HGNC:8910] | 9048 | -0.235 | -0.4834 | Yes |
| 15 | AHR | aryl hydrocarbon receptor [Source:HGNC Symbol;Acc:HGNC:348] | 9263 | -0.253 | -0.4814 | Yes |
| 16 | ATF7 | activating transcription factor 7 [Source:HGNC Symbol;Acc:HGNC:792] | 9281 | -0.254 | -0.4639 | Yes |
| 17 | CIT | citron rho-interacting serine/threonine kinase [Source:HGNC Symbol;Acc:HGNC:1985] | 9356 | -0.260 | -0.4505 | Yes |
| 18 | THRB | thyroid hormone receptor beta [Source:HGNC Symbol;Acc:HGNC:11799] | 9820 | -0.303 | -0.4642 | Yes |
| 19 | RARA | retinoic acid receptor alpha [Source:HGNC Symbol;Acc:HGNC:9864] | 10141 | -0.335 | -0.4644 | Yes |
| 20 | VGLL4 | vestigial like family member 4 [Source:HGNC Symbol;Acc:HGNC:28966] | 10338 | -0.354 | -0.4535 | Yes |
| 21 | CREBBP | CREB binding protein [Source:HGNC Symbol;Acc:HGNC:2348] | 10881 | -0.414 | -0.4651 | Yes |
| 22 | GATA1 | GATA binding protein 1 [Source:HGNC Symbol;Acc:HGNC:4170] | 10958 | -0.425 | -0.4397 | Yes |
| 23 | ZBTB17 | zinc finger and BTB domain containing 17 [Source:HGNC Symbol;Acc:HGNC:12936] | 10996 | -0.429 | -0.4109 | Yes |
| 24 | GATA2 | GATA binding protein 2 [Source:HGNC Symbol;Acc:HGNC:4171] | 11058 | -0.439 | -0.3833 | Yes |
| 25 | CDK9 | cyclin dependent kinase 9 [Source:HGNC Symbol;Acc:HGNC:1780] | 11071 | -0.441 | -0.3518 | Yes |
| 26 | PPARA | peroxisome proliferator activated receptor alpha [Source:HGNC Symbol;Acc:HGNC:9232] | 11110 | -0.445 | -0.3220 | Yes |
| 27 | MED1 | mediator complex subunit 1 [Source:HGNC Symbol;Acc:HGNC:9234] | 11146 | -0.450 | -0.2915 | Yes |
| 28 | PPARD | peroxisome proliferator activated receptor delta [Source:HGNC Symbol;Acc:HGNC:9235] | 11301 | -0.471 | -0.2687 | Yes |
| 29 | EP300 | E1A binding protein p300 [Source:HGNC Symbol;Acc:HGNC:3373] | 11741 | -0.540 | -0.2631 | Yes |
| 30 | EPAS1 | endothelial PAS domain protein 1 [Source:HGNC Symbol;Acc:HGNC:3374] | 11758 | -0.542 | -0.2244 | Yes |
| 31 | HIF1A | hypoxia inducible factor 1 subunit alpha [Source:HGNC Symbol;Acc:HGNC:4910] | 11833 | -0.556 | -0.1892 | Yes |
| 32 | TFAM | "transcription factor A, mitochondrial [Source:HGNC Symbol;Acc:HGNC:11741]" | 12175 | -0.637 | -0.1687 | Yes |
| 33 | GATA3 | GATA binding protein 3 [Source:HGNC Symbol;Acc:HGNC:4172] | 12276 | -0.670 | -0.1271 | Yes |
| 34 | MED25 | mediator complex subunit 25 [Source:HGNC Symbol;Acc:HGNC:28845] | 12480 | -0.741 | -0.0883 | Yes |
| 35 | RPL23 | ribosomal protein L23 [Source:HGNC Symbol;Acc:HGNC:10316] | 12558 | -0.777 | -0.0370 | Yes |
| 36 | RORA | RAR related orphan receptor A [Source:HGNC Symbol;Acc:HGNC:10258] | 12696 | -0.862 | 0.0159 | Yes |
Table: GSEA details [plain text format]

  

Fig 2: GOMF\_TRANSCRIPTION\_COACTIVATOR\_BINDING      
 Blue-Pink O' Gram in the Space of the Analyzed GeneSet

  

Fig 3: GOMF\_TRANSCRIPTION\_COACTIVATOR\_BINDING: Random ES distribution      
 Gene set null distribution of ES for **GOMF\_TRANSCRIPTION\_COACTIVATOR\_BINDING**
